# Supplementary material for: Inflammation and the pathological progression of Alzheimer’s disease are associated with low circulating choline levels
Source: Acta Neuropathol. 2023 Aug 7;146(4):565–83. doi: 10.1007/s00401-023-02616-7 (PMC10499952; doi:10.1007/s00401-023-02616-7)
Supplement: Supplementary file 1 — Supplementary file1 (DOCX 957 KB) [file 401_2023_2616_MOESM1_ESM.docx]

| **Last BMI** | | | | | | | | |
| --- | --- | --- | --- | --- | --- | --- | --- | --- |
| Status (*n*) | <18.5 | 18.5-24.9 | 25.0-29.9 | 30.0-34.9 | 35.0-39.9 | *M* | *SD* |  |
| CON (9) | 2 | 6 | 1 | 0 | 0 | 21.5 | 3.4 |  |
| MCI Sparse (10) | 0 | 4 | 6 | 1 | 0 | 25.3 | 3.9 |  |
| MCI High (12) | 2 | 5 | 4 | 1 | 0 | 23.5 | 3.9 |  |

| **Diagnosis Age** | | | | | | | | |
| --- | --- | --- | --- | --- | --- | --- | --- | --- |
| Status (*n*) | 70-74 | 75-79 | 80-84 | 85-89 | ≥90 | *M* | *SD* |  |
| CON (0) |  |  |  |  |  |  |  |  |
| MCI Sparse (3) | 0 | 0 | 2 | 1 | 0 | 84.0 | 3.0 |  |
| MCI High (8) | 1 | 0 | 0 | 3 | 4 | 86.3 | 6.1 |  |

| **Years Since Diagnosis** | | | | | | | | |
| --- | --- | --- | --- | --- | --- | --- | --- | --- |
| Status (*n*) | 1 | 2 | 3 | 4 | 5 | *M* | *SD* |  |
| CON (0) |  |  |  |  |  |  |  |  |
| MCI Sparse (5) | 3 | 2 | 0 | 0 | 0 | 1.4 | 0.5 |  |
| MCI High (10) | 3 | 2 | 2 | 1 | 2 | 2.7 | 1.6 |  |

|  | **Expired Age** | | | | | | | | | |
| --- | --- | --- | --- | --- | --- | --- | --- | --- | --- | --- |
| Status (*n*) | | 50-69 | 70-74 | 75-79 | 80-84 | 85-89 | ≥90 | *M* | *SD* |  |
| CON (10) | | 1 | 1 | 2 | 4 | 1 | 1 |  |  |  |
| MCI Sparse (10) | | 0 | 0 | 1 | 3 | 2 | 4 |  |  |  |
| MCI High (12) | | 0 | 0 | 1 | 1 | 1 | 6 |  |  |  |

| **Postmortem Interval (hours)** | | | | | | |
| --- | --- | --- | --- | --- | --- | --- |
| Status (*n*) | 1-3 | 4-6 | 7-9 | *M* | *SD* |  |
| CON (10) | 8 | 2 | 0 | 3.2 | 0.7 |  |
| MCI Sparse (10) | 6 | 4 | 0 | 3.2 | 1.1 |  |
| MCI High (12) | 5 | 6 | 1 | 4.1 | 1.2 |  |

| **Final MMSE** | | | | | | | | | **Months Since Last MMSE** | |
| --- | --- | --- | --- | --- | --- | --- | --- | --- | --- | --- |
| Status (*n*) | >25 | 20-24 | 15-19 | 10-14 | 5-9 | 0-4 | *M* | *SD* | *M* | *SD* |
| CON (7) | 7 | 0 | 0 | 0 | 0 | 0 | 28.3 | 1.8 | 19.6 | 19.3 |
| MCI Sparse (10) | 9 | 1 | 0 | 0 | 0 | 0 | 27.0 | 3.2 | 16.1 | 8.5 |
| MCI High (12) | 10 | 2 | 0 | 0 | 0 | 0 | 26.6 | 2.4 | 16.4 | 13.4 |

| **APOE Status** | | | | | |
| --- | --- | --- | --- | --- | --- |
| Status (*n*) | 2/2 | 2/3 | 3/3 | 3/4 | 4/4 |
| CON (9) | 0 | 0 | 8 | 1 | 0 |
| MCI Sparse (10) | 1 | 2 | 3 | 3 | 0 |
| MCI High (12) | 0 | 3 | 7 | 2 | 0 |

| **Braak staging** | | | | | |
| --- | --- | --- | --- | --- | --- |
| Status (*n*) | I | II | III | IV | V |
| CON (10) | 2 | 3 | 5 | 0 | 0 |
| MCI Sparse (10) | 0 | 4 | 6 | 0 | 0 |
| MCI High (12) | 0 | 0 | 0 | 11 | 1 |

| **CERAD neuritic plaque density** | | | | |
| --- | --- | --- | --- | --- |
| Status (*n*) | Zero | Sparse | Moderate | Frequent |
| CON (10) | 4 | 6 | 0 | 0 |
| MCI Sparse (10) | 4 | 4 | 2 | 0 |
| MCI High (12) | 0 | 0 | 0 | 12 |

| **Cerebral Amyloid Angiopathy (CAA) Status** | | | | |
| --- | --- | --- | --- | --- |
| Status (*n*) | Yes | No |  |  |
| CON (10) | 4 | 6 |  |  |
| MCI Sparse (10) | 6 | 4 |  |  |
| MCI High (12) | 9 | 3 |  |  |

| **Cerebral white matter rarefaction (CWMR)** | | | | |
| --- | --- | --- | --- | --- |
| Status (*n*) | Yes | No |  |  |
| CON (10) | 5 | 5 |  |  |
| MCI Sparse (10) | 4 | 6 |  |  |
| MCI High (12) | 11 | 1 |  |  |

| **TDP-43 Pathology** | | | |
| --- | --- | --- | --- |
| Status (*n*) | Yes | No | Undiagnosed |
| CON (5) | 0 | 3 | 2 |
| MCI Sparse (8) | 6 | 2 | 0 |
| MCI High (10) | 2 | 3 | 5 |

| **Brain Weight (grams)** | | | |
| --- | --- | --- | --- |
| Status (*n*) | *M* | SEM | Range |
| CON (10) | 1210 | 29.4 | 1002-1456 |
| MCI Sparse (10) | 1186 | 92.9 | 1060-1360 |
| MCI High (12) | 1155 | 112.4 | 928-1340 |

| **NIA-Reagan Diagnosis** | | | | |
| --- | --- | --- | --- | --- |
| Status (*n*) | No AD | Low | Intermediate | High |
| CON (10) | 10 | 0 | 0 | 0 |
| MCI Sparse (10) | 10 | 0 | 0 | 0 |
| MCI High (12) | 9 | 0 | 3 | 0 |

**Supplementary Table 1:** **MCI Human characteristic profiles**.

**
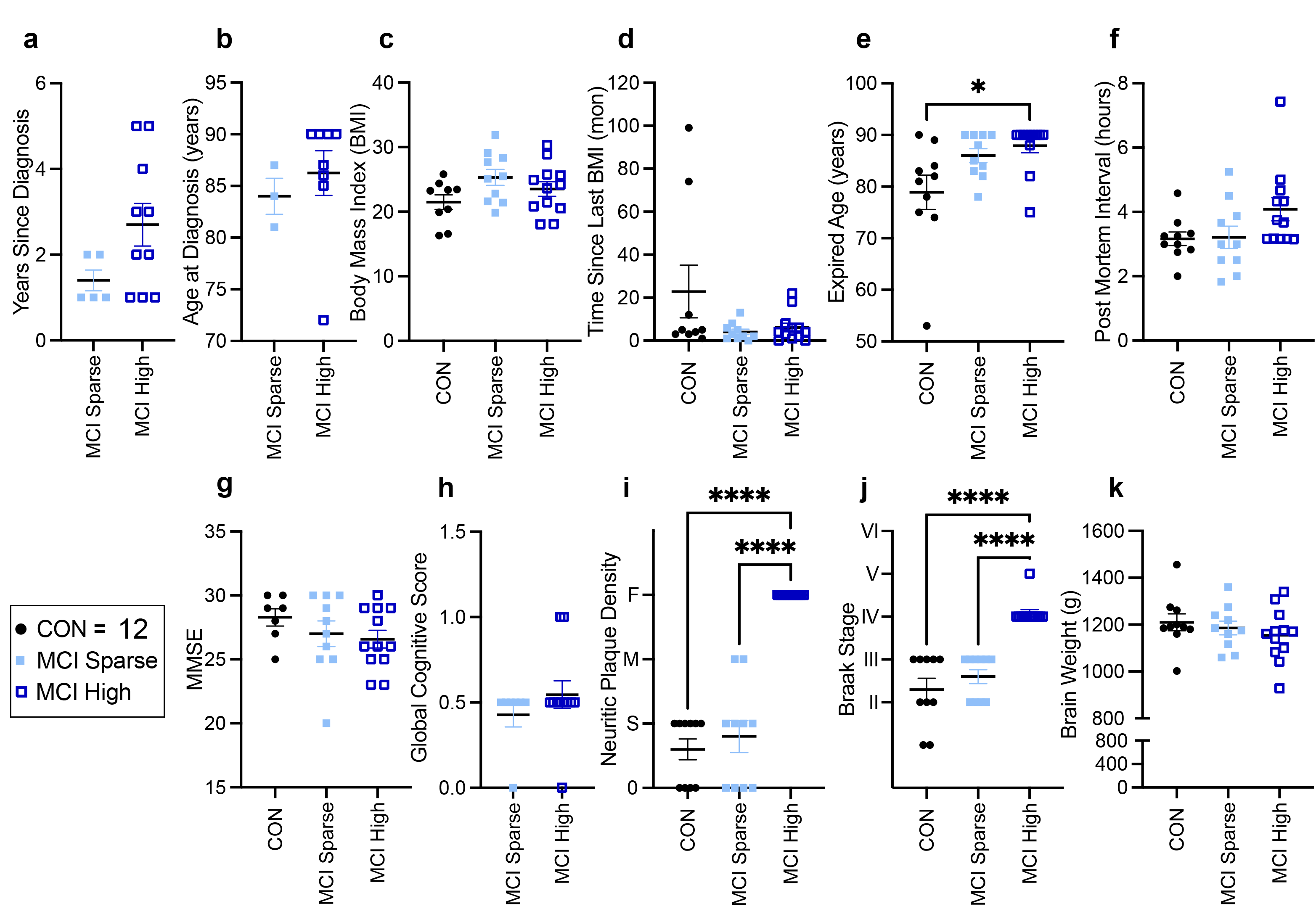
**

**Supplemental Figure 1: Graphical representation of human MCI subject profiles.** Healthy controls (n = 10), MCI with sparse pathology (MCI Sparse; sparse CERAD neuritic plaque density and Braak stage = II - III; n = 10), MCI with high pathology (MCI High; frequent CERAD neuritic plaque density and Braak stage = IV - V; n = 12). Comparison of the groups **(a)** years since diagnosis, **(b)** age at diagnosis, **(c)** BMI, **(d)** time since last BMI measurement, **(e)** expired age, **(f)** PMI, and **(g)** MMSE, and **(h)** Global cognitive score. Subjects differed from each other according to diagnosis on **(i)** neuritic plaque density. 0=Zero, S=Sparse, M=Moderate, F=Frequent, and **(j)** Braak stage, but not **(k)** brain weight. Data are reported as means ± SEM. *p<0.05, ****p<0.0001.

| **Last BMI** | | | | | | | | |
| --- | --- | --- | --- | --- | --- | --- | --- | --- |
| Status (*n*) | <18.5 | 18.5-24.9 | 25.0-29.9 | 30.0-34.9 | 35.0-39.9 | >40 | *M* | *SD* |
| CON (8) | 0 | 7 | 2 | 1 | 0 | 0 | 23.62 | 4.05 |
| AD Mod (10) | 2 | 3 | 2 | 1 | 1 | 1 | 26.25 | 8.79 |
| AD Sev (11) | 1 | 7 | 2 | 1 | 0 | 0 | 24.23 | 3.45 |

| **Diagnosis Age** | | | | | | | | |
| --- | --- | --- | --- | --- | --- | --- | --- | --- |
| Status (*n*) | 65-69 | 70-74 | 75-79 | 80-84 | 85-89 | ≥90 | *M* | *SD* |
| CON (1)* | 0 | 0 | 0 | 1 | 0 | 0 |  |  |
| AD Mod (12) | 1 | 2 | 2 | 1 | 6 | 1 | 80.83 | 8.01 |
| AD Sev (12) | 1 | 3 | 4 | 3 | 1 | 0 | 77.17 | 1.61 |

| **Years Since Diagnosis** | | | | | | | | |
| --- | --- | --- | --- | --- | --- | --- | --- | --- |
| Status (*n*) | 1-2 | 3-5 | 6-8 | 9-11 | 12-14 | 15-17 | *M* | *SD* |
| CON (1)* | 1 | 0 | 0 | 0 | 0 | 0 |  |  |
| AD Mod (12) | 1 | 5 | 4 | 0 | 1 | 1 | 6.5 | 4.36 |
| AD Sev (12) | 1 | 1 | 6 | 2 | 0 | 2 | 8.25 | 3.81 |

| **Expired Age** | | | | | | | | |
| --- | --- | --- | --- | --- | --- | --- | --- | --- |
| Status (*n*) | 70-74 | 75-79 | 80-84 | 85-89 | ≥90 |  | *M* | *SD* |
| CON (12) | 1 | 1 | 2 | 3 | 5 |  | 85 | 6.61 |
| AD Mod (12) | 0 | 2 | 1 | 4 | 5 |  | 86.17 | 4.97 |
| AD Sev (12) | 0 | 2 | 3 | 5 | 2 |  | 84.75 | 4.21 |

| **Postmortem Interval (hours)** | | | | | | | | |
| --- | --- | --- | --- | --- | --- | --- | --- | --- |
| Status (*n*) | 1-3 | 4-6 | 7-9 | 10-12 | 13-15 | 16-18 | *M* | *SD* |
| CON (12) | 10 | 2 | 0 | 0 | 0 | 0 | 2.87 | 1.26 |
| AD Mod (12) | 9 | 0 | 2 | 0 | 1 | 0 | 4.56 | 3.7 |
| AD Sev (12) | 9 | 2 | 0 | 0 | 0 | 1 | 3.92 | 4.04 |

| **Final MMSE** | | | | | | | | | **Months Since Last MMSE** | |
| --- | --- | --- | --- | --- | --- | --- | --- | --- | --- | --- |
| Status (*n*) | >25 | 20-24 | 15-19 | 10-14 | 5-9 | 0-4 | *M* | *SD* | *M* | *SD* |
| CON (8) | 8 | 0 | 0 | 0 | 0 | 0 | 28.5 | 1.6 | 20.4 | 17.3 |
| AD Mod (11) | 3 | 2 | 4 | 1 | 0 | 0 | 21.37 | 5.87 | 19.6 | 13.7 |
| AD Sev (11) | 1 | 1 | 1 | 3 | 1 | 3 | 11.8 | 3.45 | 29.9 | 35.1 |

| **APOE Status** | | | | | |
| --- | --- | --- | --- | --- | --- |
| Status (*n*) | 2/2 | 2/3 | 3/3 | 3/4 | 4/4 |
| CON (12) | 1 | 2 | 6 | 2 | 1 |
| AD Mod (12) | 0 | 2 | 6 | 4 | 0 |
| AD Sev (11) | 0 | 1 | 4 | 4 | 2 |

| **Braak staging** | | | | | |
| --- | --- | --- | --- | --- | --- |
| Status (*n*) | <II | III | IV | V | VI |
| CON (12) | 6 | 6 | 0 | 0 | 0 |
| AD Mod (12) | 0 | 0 | 12 | 0 | 0 |
| AD Sev (12) | 0 | 0 | 0 | 0 | 12 |

| **CERAD neuritic plaque density** | | | | |
| --- | --- | --- | --- | --- |
| Status (*n*) | Zero | Sparse | Moderate | Frequent |
| CON (12) | 5 | 7 | 0 | 0 |
| AD Mod (12) | 0 | 0 | 5 | 7 |
| AD Sev (12) | 0 | 0 | 0 | 12 |

| **Cerebral Amyloid Angiopathy (CAA) Status** | | | | |
| --- | --- | --- | --- | --- |
| Status (*n*) | Yes | No |  |  |
| CON (12) | 3 | 9 |  |  |
| AD Mod (12) | 7 | 5 |  |  |
| AD Sev (12) | 11 | 1 |  |  |

| **Cerebral white matter rarefaction (CWMR)** | | | | |
| --- | --- | --- | --- | --- |
| Status (*n*) | Yes | No |  |  |
| CON (12) | 6 | 6 |  |  |
| AD Mod (12) | 6 | 6 |  |  |
| AD Sev (12) | 10 | 2 |  |  |

| **TDP-43 Pathology** | | | |
| --- | --- | --- | --- |
| Status (*n*) | Yes | No | Undiagnosed |
| CON (6) | 3 | 3 | 0 |
| AD Mod (11) | 2 | 7 | 2 |
| AD Sev (9) | 1 | 6 | 2 |

| **Brain Weight** | | | |
| --- | --- | --- | --- |
| Status (*n*) | *M* | SEM | Range |
| CON (12) | 1194.75 | 30.25 | 1055-1440 |
| AD Mod (12) | 1103 | 34.97 | 960-1300 |
| AD Sev (12) | 1026.33 | 24.18 | 900-1150 |

| **NIA-Reagan Diagnosis** | | | | |
| --- | --- | --- | --- | --- |
| Status (*n*) | No AD | Low | Intermediate | High |
| CON (12) | 12 | 0 | 0 | 0 |
| AD Mod (12) | 0 | 0 | 12 | 0 |
| AD Sev (12) | 0 | 0 | 0 | 12 |
| *Mild Cognitive Impairment Diagnosis | | | |  |

**Supplementary Table 2:** **AD Human characteristic profiles**.

**
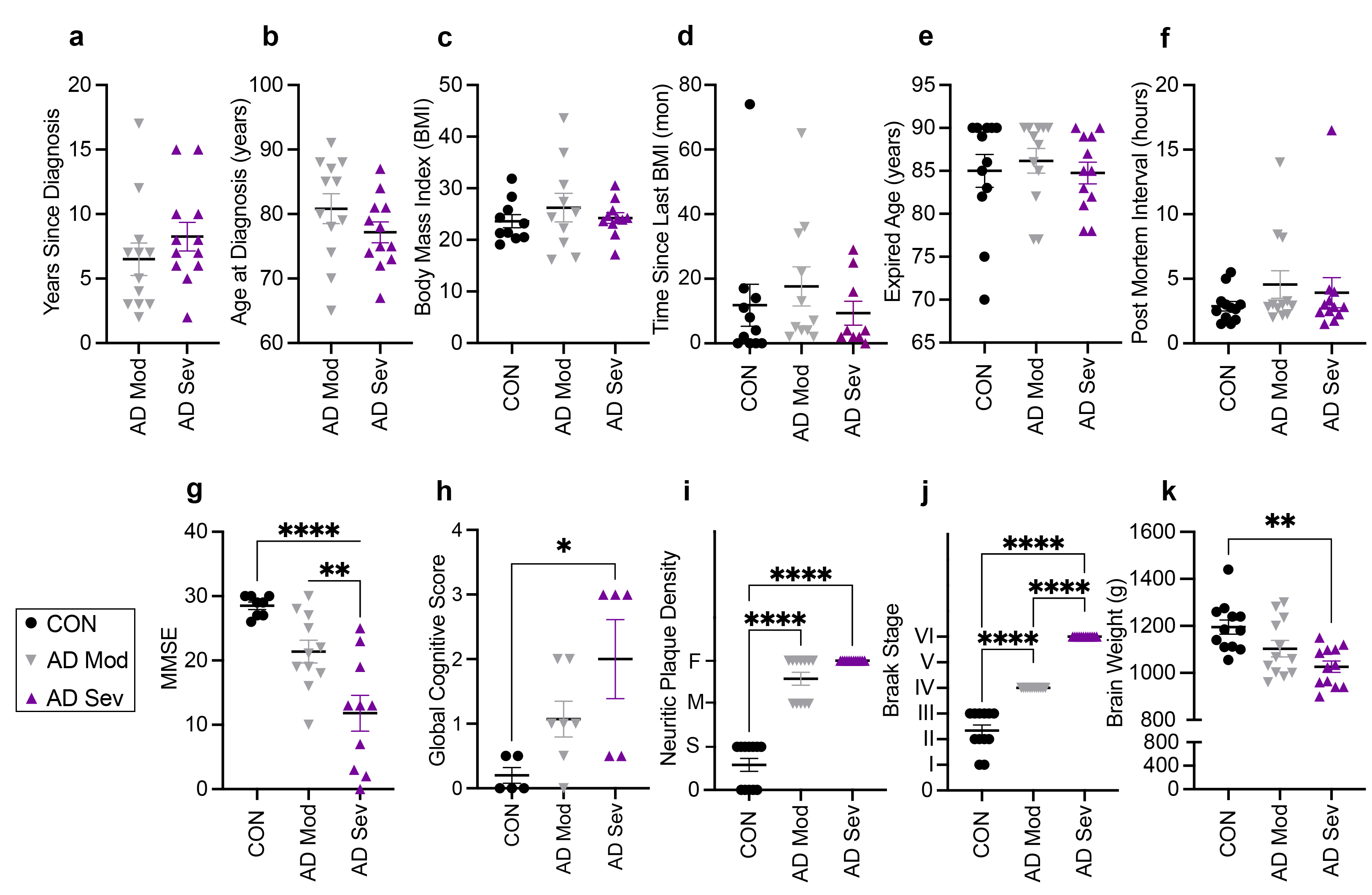
**

**Supplemental Figure 2: Graphical representation of human AD subject profiles.** Healthy controls with Braak stage ≤ III (CON, n = 12), moderate AD with Braak stage = IV and moderate to frequent CERAD neuritic plaques (AD Mod, n = 12), and severe AD with Braak stage = VI and frequent CERAD neuritic plaques (AD Sev, n = 12). Subjects were matched on **(a)** years since diagnosis, **(b)** age at diagnosis, **(c)** BMI, **(d)** time since last BMI measurement, **(e)** expired age, and **(f)** PMI. Subjects differed from each other according to diagnosis on **(g)** MMSE, **(h)** Global cognitive score, **(i)** neuritic plaque density. 0=Zero, S=Sparse, M=Moderate, F=Frequent, **(j)** Braak stage, **(k)** brain weight. Data are reported as means ± SEM. *p<0.05, **p<0.01, ****p<0.0001.

|  |  |
| --- | --- |
